# Correlation of PD-L1 and HIF-1 Alpha Expression with KRAS Mutation and Clinicopathological Parameters in Non-Small Cell Lung Cancer

**DOI:** 10.3390/cimb47020121

**Published:** 2025-02-13

**Authors:** Seda Er Özilhan, Safa Can Efil, Doğukan Çanakçı, Yetkin Ağaçkıran, Didem Şener Dede, Nilüfer Onak Kandemir, Mehmet Doğan, Tuba Dilay Kökenek Ünal, Merve Meryem Kıran, Serra Kayaçetin, Hilal Balta, Hayriye Tatlı Doğan

**Affiliations:** 1 Department of Pathology, Ankara Bilkent City Hospital, Ankara 06800, Turkey; 2 Department of Medical Oncology, Ankara Bilkent City Hospital, Ankara 06800, Turkey; 3 Faculty of Medicine, Ankara Yıldırım Beyazıt University, Ankara 06800, Turkey; 4 Department of Pathology, Ankara Atatürk Sanatoryum Hospital, Health Sciences University, Ankara 06290, Turkey; 5 Department of Medical Oncology, Faculty of Medicine, Ankara Yıldırım Beyazıt University, Ankara 06800, Turkey; 6 Department of Pathology, Faculty of Medicine, Ankara Yıldırım Beyazıt University, Ankara 06800, Turkey; 7 Department of Pathology, Ankara Bilkent City Hospital, Health Sciences University, Ankara 06290, Turkey

**Keywords:** HIF-1α, KRAS, lung cancer, PD-L1

## Abstract

**Background:** Lung cancer remains the leading cause of cancer-related deaths worldwide, with non-small cell lung carcinomas (NSCLCs) comprising the majority of cases. Among the common driver mutations, KRAS plays a critical role in guiding treatment strategies. This study evaluates the expression of programmed death-ligand 1 (PD-L1) and hypoxia-inducible factor 1-alpha (HIF-1α) in *KRAS*-mutant NSCLCs and investigates their associations with clinicopathological findings. **Methods:** A total of 85 cases with *KRAS* mutations were analyzed. Immunohistochemical staining for HIF-1α and PD-L1 was performed, and their relationships with mutation status and prognostic variables were assessed. **Results:** A significant correlation was identified between HIF-1α expression and PD-L1 expression in tumor cells. While the *KRAS* G12C mutation was not significantly associated with HIF-1α expression in tumor cells, it demonstrated a notable relationship with HIF-1α expression in the tumor microenvironment and PD-L1 expression. However, PD-L1 and HIF-1α expression did not significantly influence overall survival outcomes. **Conclusions:** Expression of PD-L1 was positively correlated with HIF-1α, which may provide evidence for a novel therapy targeting PD-L1 and HIF-1α in NSCLC. Further comprehensive studies are warranted to elucidate the prognostic implications of tumor–microenvironment and mutation interactions.

## 1. Introduction

Lung cancer remains the leading cause of cancer-related deaths worldwide, ranking first among men and second among women [1]. It is broadly categorized into small cell lung cancer (SCLC) and non-small cell lung cancer (NSCLC), with further subclassifications introduced by the World Health Organization (WHO) in 2021.NSCLC accounts for 85–90% of all lung cancers, while SCLC constitutes 10–15%. Among NSCLCs, adenocarcinomas comprise 40%, squamous cell carcinomas (SCC) 25–30%, and large cell carcinomas 10–15% of cases [2]. Due to the high incidence and frequent late-stage diagnoses of lung cancers, recent research has prioritized early detection and treatment strategies [3].

At diagnosis, metastases are present in 40% of NSCLC cases and 62% of SCLC cases [4]. Molecular advances have identified various mutations in NSCLC, paving the way for targeted therapies, particularly in metastatic cases. One of the most significant advancements has been in targeting the Kirsten rat sarcoma viral oncogene homolog (*KRAS*) gene, which plays a critical role in NSCLC, particularly adenocarcinomas. *KRAS* mutations are observed in 33% of lung adenocarcinomas, with *KRAS* G12C mutations accounting for 44% of these cases [5].

One of the critical factors driving the progression and treatment resistance of NSCLC is the tumor microenvironment, particularly the presence of hypoxia. As tumors grow, limited vascular diffusion leads to hypoxia, triggering adaptive pathways in tumor cells. Tumor cells adapt to hypoxia by activating various pathways [6]. Hypoxia-inducible factor-1α (HIF-1α), an oxygen-sensitive transcription factor, plays a central role in this adaptation. HIF-1α transcriptionally activates involved in angiogenesis, cell survival, glucose metabolism, and invasion under hypoxic conditions [7]. HIF-1α also plays a crucial role in malignant cell proliferation and is reported to induce programmed death ligand-1 (PD-L1) expression. Programmed death ligand-1 (PD-L1) is an immune checkpoint protein that suppresses anti-cancer immunity by inhibiting T-cell activity. The co-expression of PD-L1 and HIF-1α is associated with poor prognosis, as these molecules collectively promote immune evasion and tumor progression [8]. Targeted therapies such as *KRAS* G12C inhibitors, along with immune checkpoint inhibitors targeting PD-L1, have significantly advanced the treatment landscape for *KRAS*-mutant NSCLC [9]. However, the interplay between *KRAS* mutations, hypoxia-induced pathways such as HIF-1α, and PD-L1 expression remains poorly understood. Falk et al. demonstrated in vitro that hypoxia significantly increases PD-L1 expression in *KRAS* G12C and *KRAS* G12D mutant cells, highlighting the interplay between hypoxia pathways and oncogenic *KRAS* mutations. Also, there was differential activation of NF-kB, ERK, and Pi3k/Akt pathways between Kras-mutant subtypes [10]. Building on this, it is plausible that PD-L1 and HIF molecules may also exhibit specific relationships with distinct *KRAS* mutations. Understanding these interactions could be critical for guiding the development of combination therapies, such as the use of anti-PD-L1 checkpoint inhibitors alongside ERK or PI3K inhibitors, tailored to the unique molecular profiles of *KRAS*-mutant tumors.

This study aims to investigate the clinicopathological characteristics of patients with *KRAS* mutations, evaluate the expression of PD-L1 and HIF-1α in these tumors, and explore their relationship with clinicopathological findings. Understanding the interactions between these molecular pathways could provide valuable insights into tumor biology and inform the development of personalized treatment strategies for NSCLC patients.

## 2. Materials and Methods

We consecutively enrolled 85 patients over the age of 18, diagnosed with NSCLC from primary or metastatic tissue and with detected somatic *KRAS* mutations between 2022 and 2023. We collected 60 cases from biopsy samples and 25 from excision materials.

### 2.1. Preparation of Sections and Immunohistochemical Examination

For each of the 85 eligible cases, tumor tissue samples were evaluated from H&E-stained slides to prepare sections for immunohistochemical staining targeting HIF-1α and PD-L1. Tumor specimens with adequate fixation and dense viable tumor cells were selected, avoiding necrotic areas. Immunohistochemical staining was performed using a fully automated immunohistochemistry staining platform (Roche Ventana Ultra, Tucson, AZ, USA). The following antibodies were used: HIF-1α (Rabbit monoclonal, EP1215Y, Abcam (Cambridge, UK); 1:200 dilution) and PD-L1 (Mouse monoclonal, anti-PD-L1, 22C3, Dako (Santa Clara, CA, USA); 1:50 dilution). Each antibody was validated with known positive controls according to the datasheets. Negative control staining was performed during the experimental procedures to ensure the validity of the results. For IHC staining, sections were deparaffinized and rehydrated, followed by antigen retrieval in CC1 (EDTA buffer, pH 8) at 95 °C for 40 min. Primary antibodies, including HIF-1α and PD-L1, were applied to sections at 37 °C for 30 min. After washing, secondary antibodies were applied, followed by DAB (3,3′-diaminobenzidine) detection. Sections were counterstained with hematoxylin to complete the staining process.

### 2.2. Evaluation of Immunohistochemical Study Results

**Figure 1:**
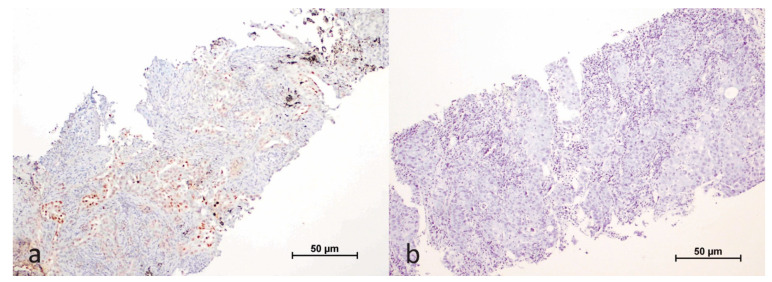
Immunohistochemical staining of HIF-1α in the nuclei of tumor cells (**a**); negative control (**b**).

We assessed the nuclear staining of the HIF-1α antibody in both tumor cells and the tumor microenvironment, which included plasma cells, macrophages, and lymphocytes. Staining in tumor cells was evaluated based on the extent of nuclear staining, expressed as a percentage. Cases with a nuclear staining rate greater than 1% in tumor cells were classified as positive (Figure 1).

**Figure 2:**
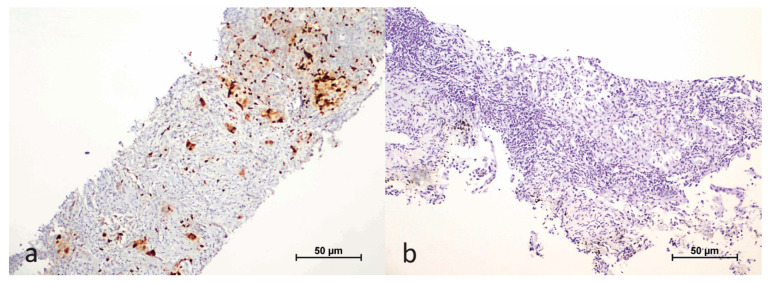
Immunohistochemical expression of HIF-1α in the tumor cells and microenvironment (**a**); negative control (**b**).

In the tumor microenvironment, we considered immune cells negative for HIF-1α antibody if they showed no staining, while any staining was regarded as positive (Figure 2).

**Figure 3:**
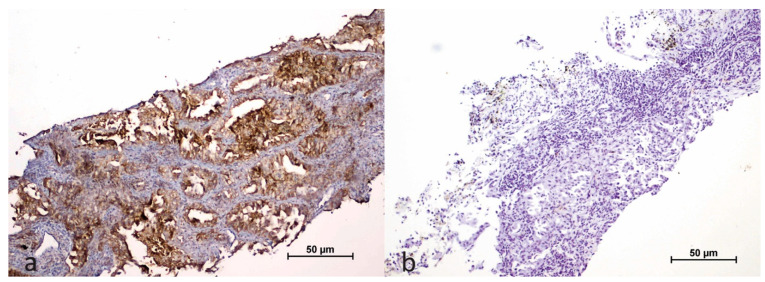
Immunohistochemical staining of PD-L1 in tumor cells (**a**); negative control (**b**).

In tumor cells, membranous PD-L1 expression was categorized as follows: negative, low expression (1–50% staining), and high expression (51–100% staining; Figure 3).

### 2.3. Molecular Analysis: Real-Time PCR Tests for KRAS

We marked the most suitable tumor area on H&E-stained slides and prepared 5 μm sections from tissue samples fixed in formalin and embedded in paraffin blocks. After manually macro-dissecting the marked areas, isolating DNA, we detected target mutations including *KRAS* codon 12, 13, 59, 61, 117, and 146 using real-time PCR reactions and analyzed results with EasyPGX^®^ software, version 4.0.13.

### 2.4. Statistical Analysis

We conducted all analyses using IBM SPSS v21.0 (IBM Corp., Armonk, NY, USA). We assessed the normality of quantitative variables with the Shapiro–Wilk test. We reported descriptive statistics as mean ± standard deviation for normally distributed variables, median (minimum-maximum) for non-normally distributed variables, and frequency (percentage) for categorical variables. We calculated survival times using the Kaplan–Meier method and examined relationships between protein expressions with Spearman correlation coefficients. To analyze non-normally distributed quantitative variables, we used the Mann–Whitney U test. We applied the chi-square or Fisher’s exact test for categorical variable analyses. We considered a *p*-value < 0.05 as statistically significant.

## 3. Results

### 3.1. Demographic Data of the Cases

In our study, the cohort consisted of 85 patients with an average diagnosis age of 66.4 years. Among them, 66 were male and 19 were female. Regarding tumor type, 70 cases were adenocarcinomas, 5 were classified as non-small cell lung carcinomas (NSCLCs), and 10 were squamous cell carcinomas. The average tumor diameter was 43.7 mm (±25.64).

In terms of disease staging, 62 patients were at stage IV, 16 at stage III, and 7 at stage II. Somatic *KRAS* codon 12 mutations (G12R/S/C/V/D/A) were detected in 59 patients, with 32 cases (37.6%) specifically involving the G12C mutation (Table 1).

### 3.2. Evaluation of KRAS Mutation Status with HIF-1α and PD-L1 Expression

*KRAS* mutation status was evaluated alongside PD-L1 expression in tumor cells and HIF-1α expression in both tumor cells and the tumor microenvironment. Among the cases, high PD-L1 expression was observed in 24 cases, low expression in 34 cases, and no expression in 27 cases. HIF-1α staining in tumor cells was present in 39 cases, while 65 cases exhibited HIF-1α positivity in the tumor microenvironment.

No significant difference was observed in PD-L1 (%) and HIF-1α (%) values between mutant *KRAS* G12C (m*KRAS* G12C) cases and other *KRAS* mutant cases (*p* > 0.05). However, PD-L1 expression status (negative, low, and high) significantly differed according to *KRAS* mutation status (*p* = 0.041) (Table 2). Binary comparison revealed that individuals with m*KRAS* G12C had a significantly higher proportion of high PD-L1 expression (43.8%) compared to those with other *KRAS* mutations (18.9%). No significant relationship was identified between HIF-1α expression and *KRAS* mutation status (*p* = 0.759).

A positive and significant correlation was observed between PD-L1% and HIF-1α% expression in tumor cells, irrespective of *KRAS* mutation status (Spearman r = 0.321; *p* = 0.003).

**Figure 4:**
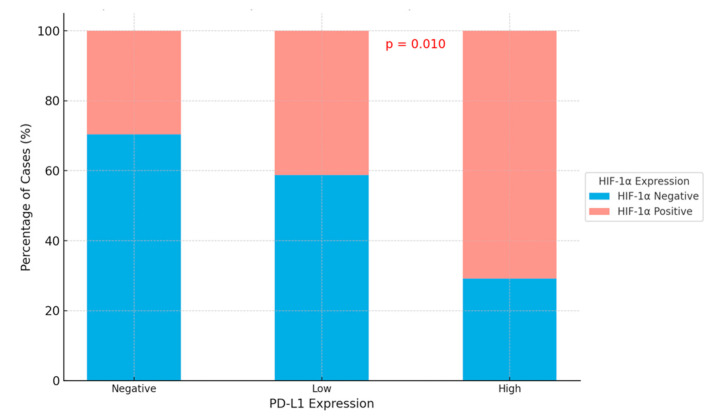
The bar chart illustrates the relationship between HIF-1α expression (negative or positive) and PD-L1 expression levels (negative, low, and high) in tumor cells. A notable correlation is observed between increased HIF-1α positivity and higher levels of PD-L1 expression.

In addition, cases with high PD-L1 expression rates were more frequently associated with HIF-1α-positive tumors, while PD-L1-negative tumors were more commonly associated with HIF-1α-negative cases (*p* = 0.01) (Table 3; Figure 4).

**Figure 5:**
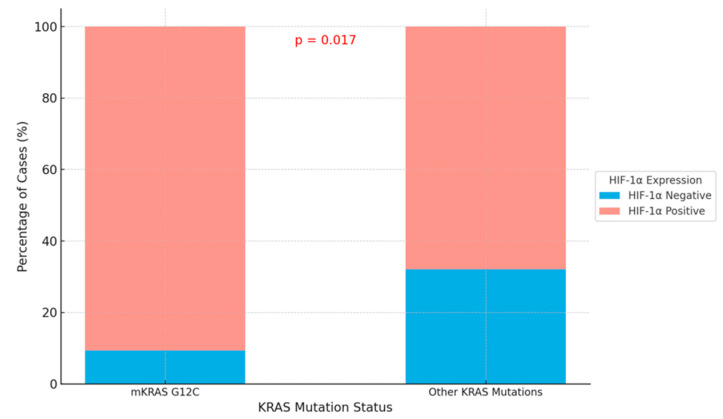
This stacked bar chart displays the percentage of cases with HIF-1α expression (positive or negative) in the tumor microenvironment, stratified by *KRAS* mutation status. A statistically significant difference (*p* = 0.017) is observed in HIF-1α expression between the mKRAS G12C and other *KRAS* mutations group.

We identified a significant difference in HIF-1α expression in the tumor microenvironment between the m*KRAS* G12C group and other *KRAS* mutation groups (*p* = 0.017) (Table 4; Figure 5). In the m*KRAS* G12C group, 90.6% of cases exhibited HIF-1α positivity in the tumor microenvironment.

### 3.3. Relationship Between HIF-1α in Tumor Microenvironment and PD-L1

**Figure 6:**
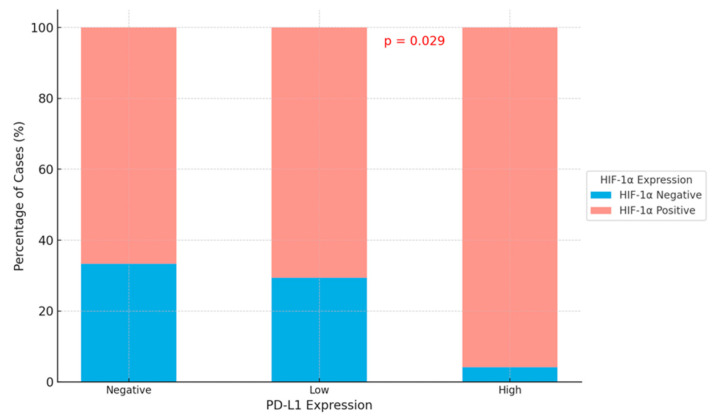
This stacked bar chart shows the percentage of cases with HIF-1α expression (positive or negative) in the tumor microenvironment, categorized by PD-L1 expression levels (negative, low, and high). A statistically significant difference (*p* = 0.029) in HIF-1α expression across PD-L1 expression levels is observed.

A significant difference was observed in the negative, low, and high expression status of PD-L1 based on the HIF-1α expression status in the tumor microenvironment (*p* = 0.029). Among cases with high PD-L1 expression, 95.8% exhibited HIF-1α positivity in the tumor microenvironment, while 4.2% were negative (Table 5; Figure 6).

### 3.4. Correlation with Prognostic Factors

*KRAS* G12C mutation was found in 28 of the 66 males and 4 of the 19 females without significant difference (*p* = 0.09) There was no significant impact of PD-L1 expression, HIF-1α expression, or their co-expression on histological type, clinical stage, or gender (all *p* > 0.05).

**Figure 7:**
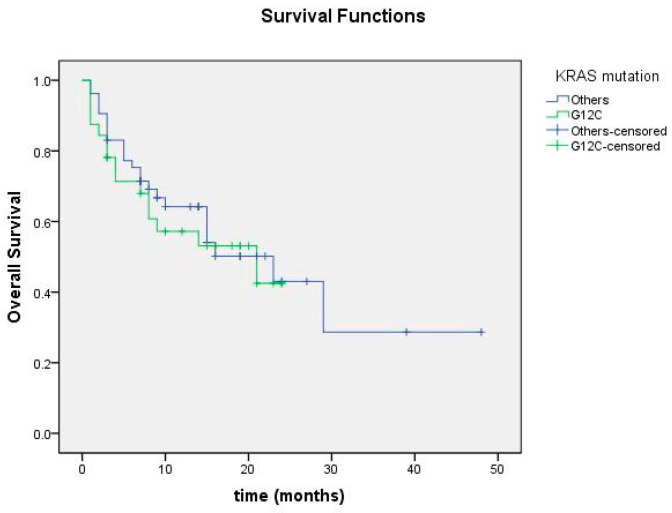
Overall survival curve according to KRAS mutation status.

Overall survival was calculated as the time from the date of initial diagnosis to the date of death or last follow-up. No significant difference was found in overall survival between m*KRAS* G12C and other *KRAS* mutation-positive groups (*p* = 0.696; Figure 7).

**Figure 8:**
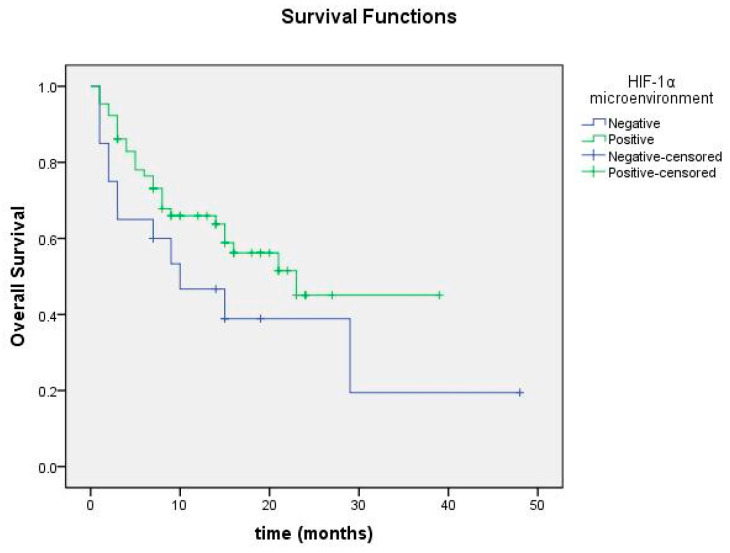
Overall survival curve according to HIF-1α expression status in tumor microenvironment.

**Figure 9:**
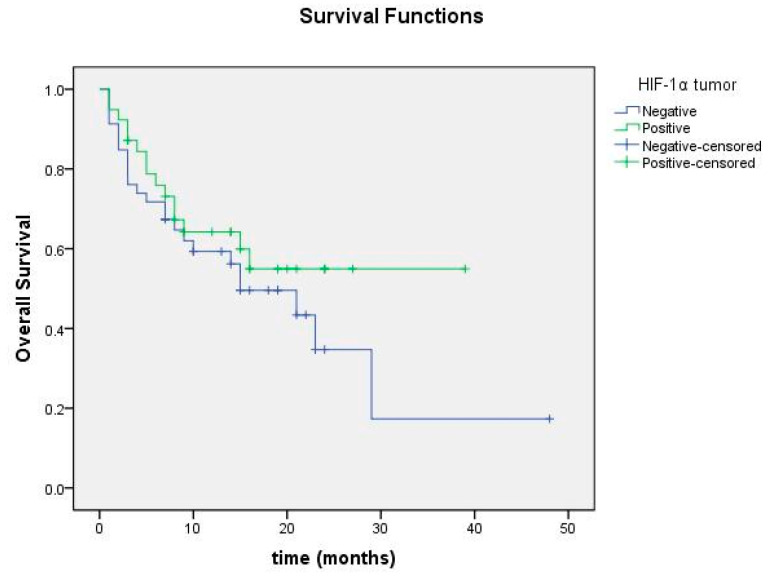
Overall survival curve according to HIF-1α expression status in tumor cells.

Overall survival was 23.09 months in the group with positive HIF-1α in the tumor microenvironment and 18.40 months in the group with negative HIF-1α, but no significant difference was found (*p* = 0.138; Figure 8). Among cases with HIF-1α positivity and negativity in tumor cells, no significant difference in overall survival was detected (Figure 9).

**Figure 10:**
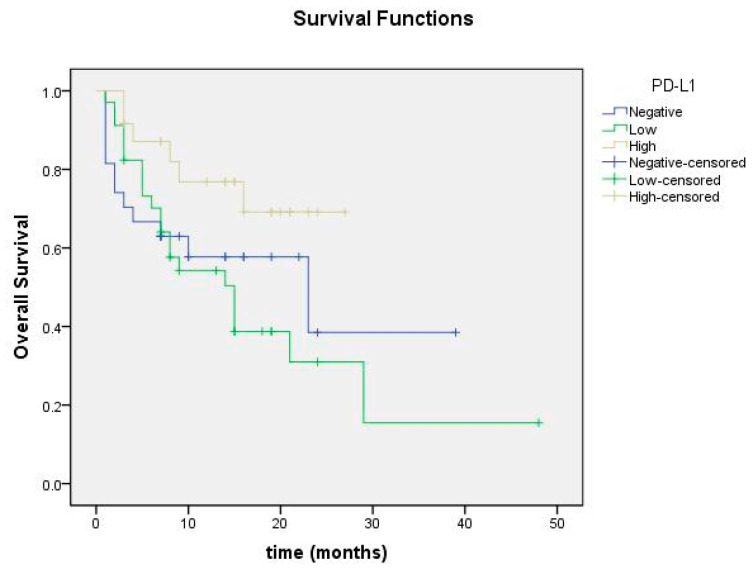
Overall survival curve according to PD-L1 expression status.

Overall survival was 21.2 months in the PD-L1 high group, 18.14 months in the low group, and 20.80 months in the negative group, with no statistically significant difference (*p* = 0.072; Figure 10).

**Figure 11:**
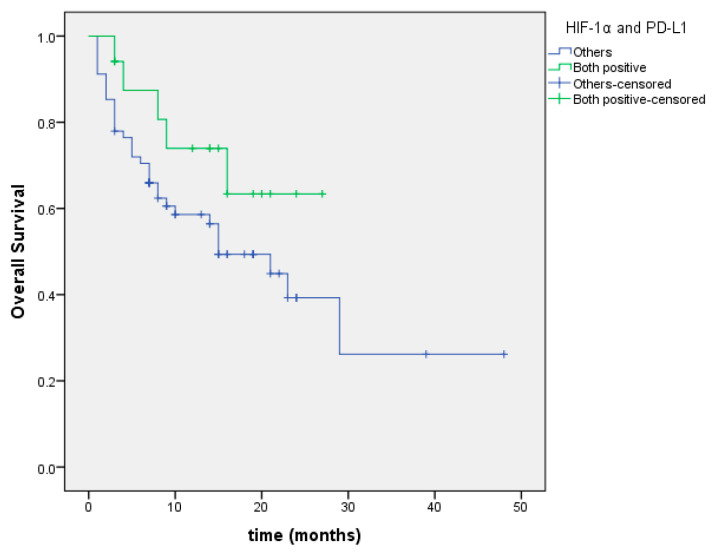
Overall survival curve based on the combined status of HIF-1α positivity and high PD-L1 expression.

Overall survival was 20.4 months in the group with both HIF-1α positivity and high PD-L1 expression, and 21.7 months in the group with both HIF-1α negativity and low or negative PD-L1 expression, with no statistically significant difference observed between the groups (*p* = 0.154; Figure 11).

## 4. Discussion

Non-small cell lung cancer (NSCLC) remains the most prevalent type of lung cancer worldwide, with adenocarcinoma as the most common subtype, accounting for 40% of cases [5]. *KRAS* mutations, particularly prevalent in NSCLC, are observed in approximately 20–22% of patients. In our study, the average patient age was 66.4, consistent with previous reports indicating an average age range of 64–66 for *KRAS* mutant lung cancers [11,12]. However, compared to another study where the male gender ratio was 47.1%, our study observed a significantly higher male gender ratio of 77.6% [13].

Survival outcomes in NSCLC are highly variable, influenced by tumor histology, treatment responses, and resistance mechanisms. Due to its high frequency and the fact that most cases are diagnosed at advanced stages, NSCLC is a key focus of research. Recent advances in identifying molecular mutations, such as *KRAS* G12C, have revolutionized treatment approaches, particularly with the advent of targeted therapies. These treatments stand out in the treatment of advanced or metastatic lung cancers. For instance, sotorasib, a *KRAS* G12C inhibitor, has shown promising results in advanced or metastatic NSCLC, marking a significant leap in mutation-specific therapy [14,15].

In our study, high PD-L1 expression was significantly more frequent in *KRAS* G12C mutant patients compared to those without the mutation. Janne et al. reported that 40.5% of *KRAS* G12C mutant patients lacked PD-L1 expression, while 23.3% had 1–49% expression, and 10.3% had 51–100% expression [15]. A meta-analysis including 4352 NSCLC patients showed no statistically significant survival difference between patients with *KRAS* G12C and non-*KRAS* G12C mutations [16].

Progression-free survival is reported to be longer in patients with low PD-L1 expression than in patients with high PD-L1 expression [17,18]. However, some studies also have conflicting data indicating that PD-L1 expression has no effect on survival or provides better survival [19,20]. Similarly, our study found no statistically significant effect of PD-L1 expression on overall survival.

While overexpression of HIF-1α is generally associated with poor treatment outcomes and increased mortality, some studies suggest it may have protective effects in surgically treated NSCLC patients. In our study, HIF-1α expression in tumor cells showed no significant relationship with *KRAS* G12C mutation status. However, a significant association was observed between *KRAS* G12C mutations and HIF-1α expression in the tumor microenvironment, emphasizing the role of the tumor microenvironment in modulating disease progression.

HIF-1α, a critical regulator of cellular responses to hypoxia, is often overexpressed in cancer due to genetic alterations such as gain-of-function mutations in oncogenes such as the *KRAS* mutation and loss-of-function mutations in tumor suppressor genes. Overexpression of HIF-1α leads to decreased sensitivity to treatment and is associated with increased mortality [7]. However, one study reported that HIF-1α overexpression may protect the survival of NSCLC patients undergoing surgical treatment. Another study found no significant difference in overall or progression-free survival according to HIF-1α expression levels [21]. Hypoxia is a characteristic finding, especially in solid tumors, due to insufficient angiogenesis in the microenvironment. Therefore, in our study, HIF-1α was examined in both tumor tissue and microenvironment. While no statistically significant relationship was found between *KRAS* G12C mutation status and HIF-1α expression in tumor cells, a significant relationship was found in terms of expression of HIF-1α in tumor microenvironment and *KRAS* G12C mutation.

The interplay between PD-L1 and HIF-1α has been increasingly recognized as a potential determinant of NSCLC prognosis. Zheng et al. reported a positive correlation between PD-L1 and HIF-1α in NSCLC, identifying both as independent poor prognostic factors [8]. Our study corroborated this positive correlation, suggesting that combined evaluation of PD-L1 and HIF-1α expression could provide valuable insights into tumor biology and treatment strategies. This information also points to the potential of developing combination therapies targeting both PD-L1 and HIF-1α.

With the growing diversity of therapeutic options, including mutation-specific treatments and immunotherapies, understanding the molecular characteristics of NSCLC tumors has become imperative. The success of immunotherapy in urological or dermatological malignancies has accelerated studies on the applicability of this treatment in lung cancer [22,23]. Immunotherapies targeting immune checkpoints via PD-L1 have shown promising results, especially in metastatic NSCLC patients [24]. For this reason, PD-L1 expression in tumor tissue and the immunotherapy applied accordingly is of great importance in survival [25,26]. Targeting immune checkpoints such as PD-L1 has proven effective in metastatic NSCLC, particularly in patients with high PD-L1 expression (≥50%). In these cases, immunotherapy monotherapy is often preferred. For patients with lower PD-L1 expression (<50%), combined chemo–immunotherapy offers superior outcomes [27,28,29]. Notably, sotorasib has demonstrated efficacy in *KRAS* G12C mutant patients with negative or low PD-L1 expression, suggesting its superiority over immunotherapy or chemotherapy in this subset [30].

Cell culture studies also report that HIF inhibition may be effective in the treatment of *KRAS* mutant colon cancer [31]. These findings, combined with the demonstrated correlation between PD-L1 and HIF-1α in NSCLC, underscore the importance of investigating combination therapies that target both pathways. Understanding tumor–immune system interactions and the molecular landscape of NSCLC will not only enhance prognosis but also guide the development of personalized, combination treatment strategies.

This study has some limitations that should be acknowledged. The relatively small cohort size of 85 patients may limit the generalizability of the findings and the statistical power to detect subtle differences across subgroups. The retrospective nature of the study may introduce selection bias and limit the ability to establish causation between observed molecular interactions and clinical outcomes. Variations in treatment regimens, including immunotherapy and targeted therapy, could have influenced survival outcomes and introduced variability into the analysis.

Future studies addressing these limitations, with larger, multicenter cohorts and prospective designs, are needed to validate our findings and further investigate the biological mechanisms underlying these molecular interactions. Experimental studies exploring the therapeutic potential of targeting PD-L1 and HIF-1α in combination are also warranted to enhance personalized treatment strategies for NSCLC patients.

## 5. Conclusions

In conclusion, our study provides valuable insights into the interplay between PD-L1 expression, HIF-1α expression, and *KRAS* mutation status in non-small cell lung cancer (NSCLC), particularly in *KRAS* G12C mutant cases. We observed a significant relationship between PD-L1 and HIF-1α expression, as well as between *KRAS* G12C mutations and HIF-1α expression in the tumor microenvironment. These findings emphasize the importance of the tumor microenvironment in influencing disease progression and suggest the potential utility of combined therapeutic approaches targeting PD-L1 and HIF-1α.

While PD-L1 expression showed no significant effect on overall survival in our cohort, its interplay with HIF-1α highlights a complex molecular interaction that could guide future treatment strategies. The demonstrated efficacy of targeted therapies such as sotorasib in *KRAS* G12C mutant cases with low or negative PD-L1 expression further underscores the importance of personalized medicine in NSCLC. The findings from this study advocate for further exploration of combination therapies targeting hypoxia pathways and immune checkpoints to improve patient outcomes.

## Figures and Tables

**Table 1:** Clinicopathological characteristics of patients.

Age of Diagnosis	66.40 ± 8.62
Gender	
Women	19 (22.4%)
Men	66 (77.6%)
Tumor type	
Adenocarcinoma	70 (82.4%)
NSCLC, NOS	5 (5.9%)
Squamous Cell Carcinoma	10 (11.8%)
*KRAS* mutation type	
G12C	32 (37.6%)
G12X	27 (31.8%)
G13D	5 (5.9%)
A146X	9 (10.6%)
Q61X	12 (14.1%)
Stage	
II	7 (8.2%)
III	16 (18.8%)
IV	62 (72.9%)
Current status	
Alive	46 (54.1%)
Exitus	39 (45.9%)

NSCLC, NOS: non-small cell lung carcinoma, diagnosis not specified.

**Table 2:** Relationship between KRAS mutation status and PD-L1, HIF-1α expressions.

	*KRAS* Mutation		*p*-Value
	m*KRAS* G12C n (%)	Other *KRAS* Mutations n (%)	
PD-L1 %	19 (0–90) 33.94 ± 35.10	4 (0–90) 18.42 ± 25.14	0.789
PD-L1 expression			
Negative	9 (28.1)	18 (34.0)	0.041
Low	9 (28.1)	25 (47.2)	
High	14 (43.8)	10 (18.9)	
HIF-1α Tumor %	0 (0–70) 6.66 ± 15.120	(0–50) 3.96 ± 9.12	0.099
HIF-1α Tumor			
Negative	18 (56.3)	28 (52.8)	0.759
Positive	14 (43.8)	25 (47.2)	

Values are summarized by median (minimum; maximum) or frequency (column percentage). Pearson chi-square test was used for comparisons between categorical variables, and the Mann–Whitney U test was used for comparisons of numerical values.

**Table 3:** Relationship between HIF-1α expression and PD-L1 expression status in tumor cells.

	HIF-1α in Tumor Cells		*p*-Value
	Negative	Positive	
PD-L1 expression			
Negative	19 (41.3)	8 (20.5)	0.010
Low	20 (43.5)	14 (35.9)	
High	7 (15.2)	17 (43.6)	

We summarized values by frequency (column percentage) and used the Pearson chi-square test to compare categorical variables.

**Table 4:** Relationship between *KRAS* mutation status and HIF-1α expression in the tumor microenvironment.

	*KRAS* Mutation		
HIF-1α Microenvironment	mKRAS G12C n (%)	Other *KRAS* Mutations n (%)	*p*-Value
Negative	3 (9.4)	17 (37.1)	0.017
Positive	29 (90.6)	36 (67.9)	

**Table 5:** Relationship between HIF-1α tumor microenvironment and PD-L1 expression.

	HIF-1α Tumor Microenvironment		*p*-Value
	Negative	Positive	
PD-L1 expression			
Negative	9 (33.3)	18 (66.7)	0.029
Low	10 (29.4)	24 (70.6)	
High	1 (4.2)	23 (95.8)	

## Data Availability

The datasets used and/or analyzed during the current study are available from the corresponding author upon reasonable request.
